# Supplementary material for: The photospheric origin of the Yonetoku relation in gamma-ray bursts
Source: Nat Commun. 2019 Apr 3;10:1504. doi: 10.1038/s41467-019-09281-z (PMC6447599; doi:10.1038/s41467-019-09281-z)
Supplement: Supplementary file 1 — Supplementary Information [file 41467_2019_9281_MOESM1_ESM.pdf]

Supplementary Information

The photospheric origin of the Yonetoku relation in gamma-ray bursts

Ito et al. 2019

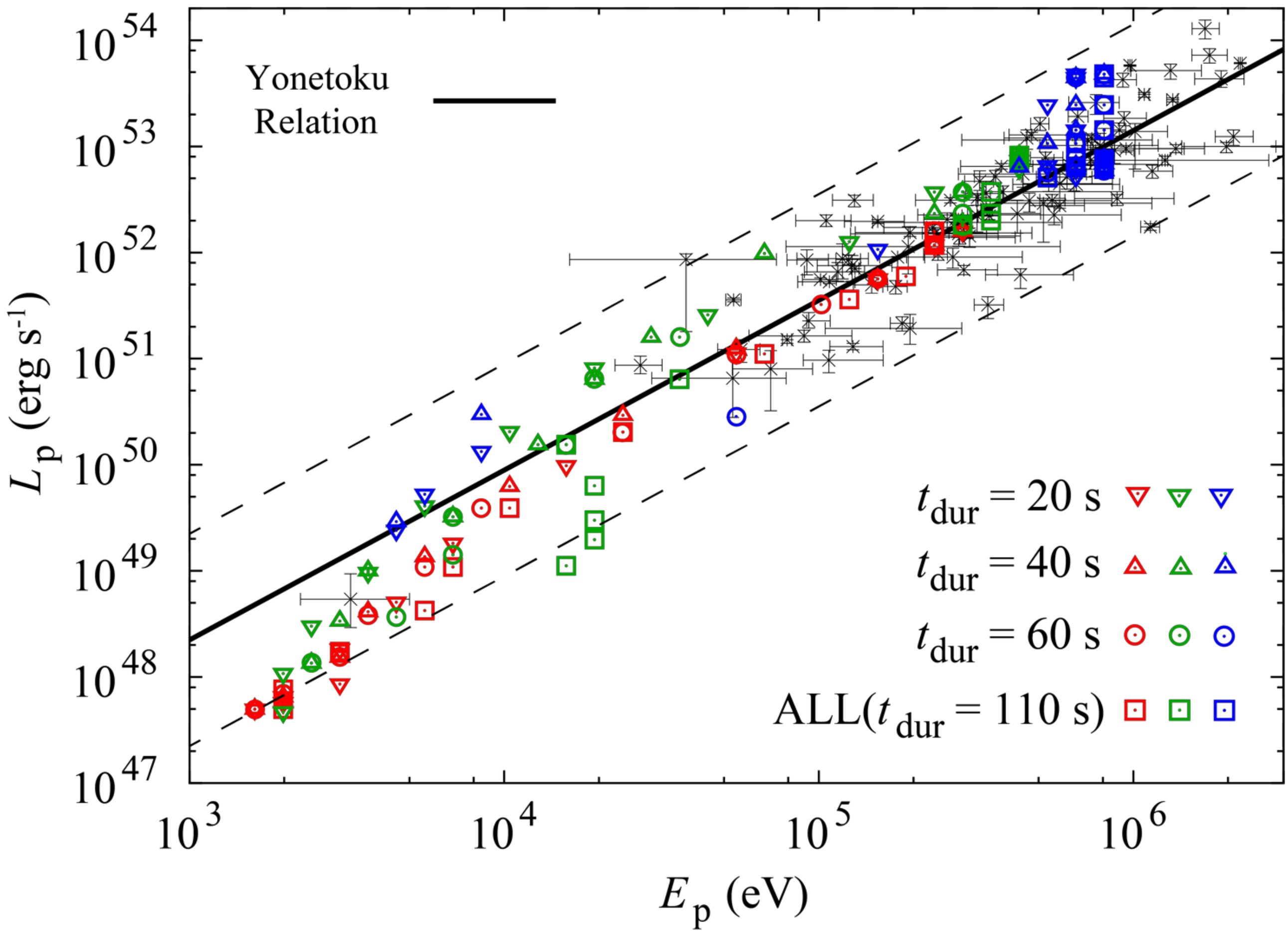

Supplementary Figure 1| Relation between spectral peak energy E_p and peak luminosity L_p. Same as Figure 3, but for the simulations with a modified prescription for the evaluation of temperature. The error bars of the observational data indicate 1-σ standard error for both E_p and L_p.

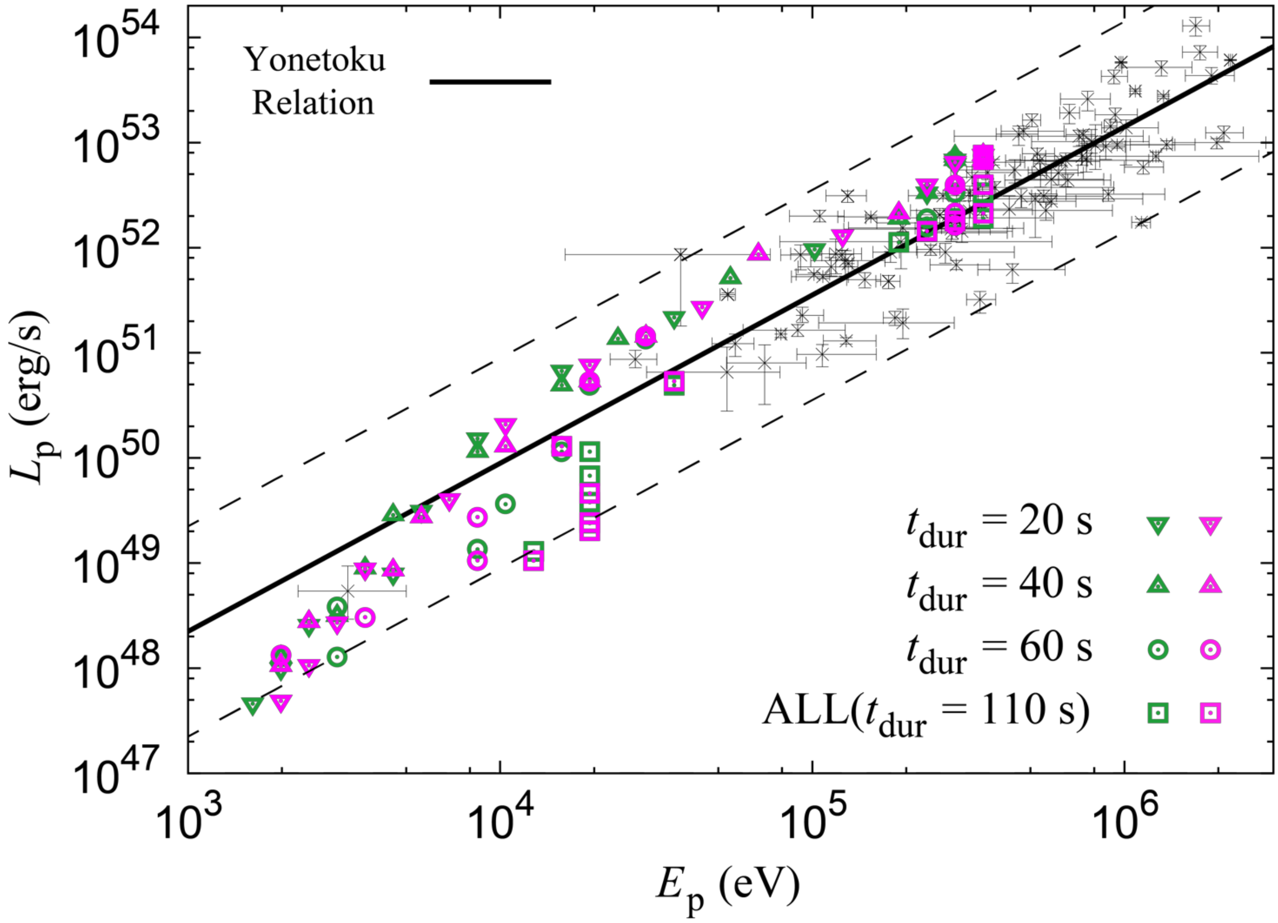

Supplementary Figure 2| Dependence of E_p and L_p on the location of photon injection. Same as Figure 3, but results for photon injection location at $\tau = 500$ (magenta) are shown with the original results which impose $\tau = 100$ (green) for the injection location. Only the $L_j = 10^{50}$ erg s^{-1} model is shown. The error bars of the observational data indicate 1-σ standard error for both E_p and L_p.

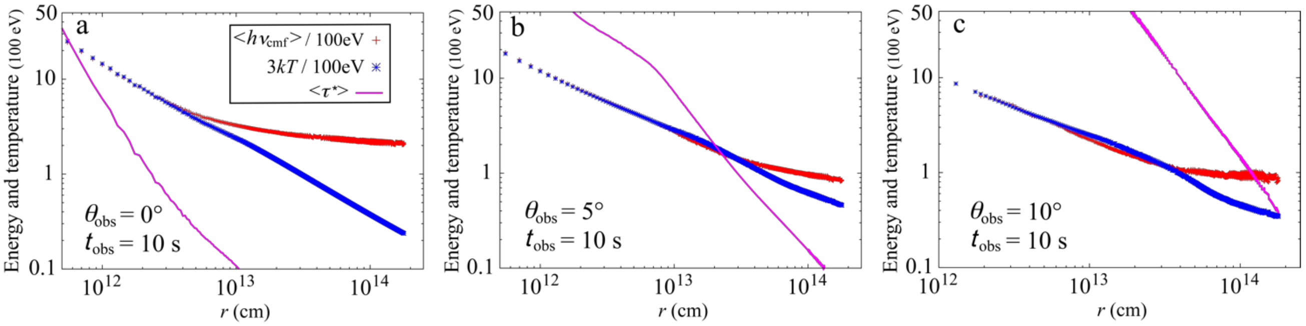

Supplementary Figure 3| Radial profile of average comoving photon energy and temperature. The red symbols display the average comoving energy of photons computed along the radial ray that corresponds to observer time $t_{obs} = 10$ s for three different viewing angles: **a** $\theta_{obs} = 0°$. **b** $\theta_{obs} = 5°$. **c** $\theta_{obs} = 10°$. The blue symbols represent the comoving temperature of fluid with which the photons interact. The magenta lines show the proxy of optical depth computed as $<\tau^*> = \Gamma(1-\beta\cos<\theta_v>)n_e\sigma_T r$, where $<\theta_v>$ is the average angle between the direction of photon propagation direction and the velocity.
